# Assessing Healthcare Access in Rural Algeria: A Survey‐Based Cross‐Sectional Analysis of Healthcare Utilization Based on Socioeconomic and Health Factors, Experiences, and Spatial Disparities

**DOI:** 10.1002/hsr2.71565

**Published:** 2025-11-23

**Authors:** Mohamed Amine Haireche, Russell Kabir, Md. Golam Kibria, Ali Davod Parsa

**Affiliations:** ^1^ Scientia Vallis Paris France; ^2^ School of Allied Health, Faculty of Health, Medicine and Social Care Anglia Ruskin University UK; ^3^ Centre for Development Action Dhaka Bangladesh

**Keywords:** Algeria, Health Services Accessibility, Health Services Utilization, Healthcare Disparities, Rural Population, Socioeconomic Factors

## Abstract

**Background and Aims:**

Equitable access to healthcare constitutes a fundamental aspect of the Algerian health policy. Yet, no studies addressed determinants of healthcare access in rural areas. This study quantified healthcare utilization (HCU) in a remote Algerian village, and analyzed the associated socioeconomic and health‐related factors, spatial disparities, and experiences with local healthcare services.

**Methods:**

A survey‐based, cross‐sectional study was conducted among 400 adult residents of Boussemghoun village, located in the Saharan Atlas Mountains. A structured questionnaire quantified the utilization of 10 basic healthcare services over the past 12‐months. Socioeconomic and health‐related factors, spatial disparities, and experiences with local healthcare services were analyzed as the independent factors of high HCU using multivariate logistic regression.

**Results:**

Among the participants, 39.7% had at least one chronic disease and 36.5% declared having traveled for healthcare during the past year. Levels of HCU were low across all care services, with the median (P75) HCU score was 4 (9) out of 33. Independent factors of high HCU included female (OR = 2.62, *p* = 0.003), widowed status (OR = 4.65, *p* = 0.026), high income (OR = 3.64, *p* = 0.048), taking 2+ chronic medications (OR = 3.61, *p* = 0.001), acute health issue in the past year (OR = 3.19, *p* = 0.013), interregional healthcare mobility (OR = 1.87, *p* = 0.041), longer travel times to local facilities (OR = 3.84, *p* = 0.004).

**Conclusion:**

Healthcare access in Boussemghoun is challenged by a critical lack of comprehensive care, resulting in frequent interregional healthcare mobility and a mismatch between HCU and apparently high healthcare needs. Strategic action is needed to mitigate the resulting inequalities and enhance local access to multidisciplinary quality care.

## Summary

- *What is known:* Rural populations often face barriers to healthcare access, but data from remote Algerian settings remain scarce.
- *What is new:* This study provides the first quantification of healthcare utilization in a remote Algerian village and identifies socioeconomic, health‐related, and spatial determinants of access.
- *Clinical implications:* Findings highlight the need for targeted policies to reduce interregional healthcare mobility and improve local access to multidisciplinary care in underserved rural areas.

## 1 Introduction

Equitable access to healthcare implies a comprehensive care supply, considering individual needs and capabilities to seek and utilize care [1]. Several countries, including low‐and middle‐income countries, have successfully improved health access among their populations [2, 3]. Despite these improvements, populations in remote areas continue to face significant challenges in accessing adequate care due to a lack of comprehensive local care delivery, and geographical and infrastructural barriers. These obstacles impede effective healthcare utilization (HCU), leading to inequalities in health outcomes and substantial financial burdens [4, 5, 6, 7, 8].

In Algeria, equitable access to healthcare constitutes a fundamental aspect of health policy. The Algerian healthcare system, a vital and primarily publicly funded social sector, aims at a universal, equitable, and free access to care [9]. Despite reform efforts, persistent disparities in resource distribution and workforce allocation continue to hinder access and HCU [10, 11, 12].

Additionally, there is scarce data addressing healthcare accessibility from a region‐specific perspective in Algeria. A review article by Aissaoui analyzed the situation of care supply in Algeria. Authors highlighted significant cross‐region disparities in healthcare provision, notably between the Northern and Southern/Saharan Provinces, impacting the cost‐effectiveness of the national health system [11]. Another study by Lahmar et al. observed disparities in healthcare accessibility within the region of Batna, attributed to the unequal distribution of healthcare facilities and a deficit in spatial infrastructure. These inequalities impacted the effectiveness of prevention programs and healthcare quality indicators in the region [10]. However, no studies have explored the specific determinants of HCU in remote Algerian villages.

The present study assessed access to healthcare among a remote Saharan village in Algeria, by quantifying the levels of HCU and analyzing the associated inequalities based on socioeconomic and health‐related factors, spatial disparity and experiences with local healthcare services. While the HCU serves as an indirect indicator for healthcare access, analyzing the determinants provides more accurate insights by highlighting interpersonal disparities in terms of needs, and predisposing and enabling factors [13]. Such data enables the development of more targeted strategies to improve healthcare access and health outcomes in underserved regions.

## 2 Project Design and Methods

### 2.1 Design and Setting

A survey‐based, cross‐sectional study was conducted in Boussemghoun village, from 20 to 22 October 2023. The village is situated in El Bayedh Province, approximately 700 kilometers southwest of Algiers, nestled by the Saharan Atlas Mountains. It accounts approximately 5000 inhabitants including a number of nomadic families in the peripheries. Additionally, Boussemghoun is distinguished by its numerous historical and touristic sites.

### 2.2 Participants

We included adult (aged ≥ 18 years) individuals residing in Boussemghoun for at least 12 months, encompassing both intramural residents and members of nomadic tribes. Tourists and non‐permanent residents were excluded. Furthermore, to ensure reliability of self‐reported data and informed consent, we excluded individuals with cognitive or sensorial disabilities that impede effective communication.

### 2.3 Sampling

The sample size was calculated to detect an unknown proportion (*P* = 50%) of participants with high HCU level, with 95% confidence interval (95% CI), 80% statistical power, and 0.05 margin error, among a finite population of 5000. The following formula was used:

$$n=\frac{N\times z^{2}\times P\times \left(1-P\right)}{\left(N-1\right)\times e^{2}+z^{2}\times P\times \left(1-P\right)}$$

Where:

- *z* is the *z*‐score (1.96 for 95% confidence)
- *P* is the estimated proportion (0.5 for 50%)
- (1 − P) is the complement of *P* (0.5)
- e is the margin error (0.05)
- N is the population size (5000)

The calculated sample size (*N* = 357) was increased by 15% (*N* = 410) to compensate for eventual incomplete or invalid participations. A convenience sampling method was used to include all eligible participants until reaching the target sample size.

### 2.4 Tool

A structured and anonymized questionnaire was designed by the author to collect the study data (Supporting Information: Appendix). It comprised five parts:

− Part 1 collected socioeconomic data such as participants' sex, age, educational level, etc.
− Part 2 explored health‐related data, such as health insurance coverage, chronic diseases, and significant health conditions during the past 12 months, etc.
− Part 3 used a 10‐item, Likert‐type agreement scale (1 = disagree to 5 = agree) to capture experiences with local healthcare services, deriving from the 5 dimensions of healthcare access including availability, accessibility, accommodation, affordability, and acceptability [14]. Items were phrased to express a positive experience regarding specific aspects of each of the 5 access dimensions, such as “I find all healthcare services and resources I need in my area”, “Transportation to healthcare facilities is reliable and accessible”, etc.
− Part 4 assessed spatial disparities including travel time and distance to the nearest primary care facility, as well as the extent of geographical barriers (e.g. mountains, rivers) and infrastructures limitations between home and the nearest primary care center.
− Finally, Part 5, assessing HCU, quantified the frequency of utilization (0 = never, 1 = 1–2 times, 2 = 3 times or more) of 10 basic healthcare services such as primary care visit, lab testing, dental care, etc., over the past 12 months. A specific item was added for Obe‐gyn, applicable only for women.

The questionnaire was redacted in plain Arabic, using simple terms tailored to the local culture and dialectal nuances. It underwent a pilot testing phase among 5 potential participants, to ensure clarity and comprehension; these were not included in the final analysis. Based on the feedback, modifications were made to the questionnaire.

### 2.5 Procedure

Data was collected during a 3‐day, 3‐arm public health action, conducted with the collaboration of a medical association and the Red Crescent division of Boussemghoun. It encompassed an awareness campaign for cardiovascular diseases, a screening campaign for hypertension, and the present study.

Data collection team encompassed 13 members, 8 from the medical association, 4 local members of the Red Crescent, and the researcher. A training session was held among all team members to standardize practices and ensure accurate understanding of the study objectives and questionnaire items. Subsequently, the team was divided into two groups: an office‐based group operating at a polyvalent facility provided by the local authorities; and a mobile team to reach potential participants in their daily life settings such as households, market places, touristic sites, etc. This approach was particularly effective in recruiting female participants, who have limited outdoor activity due to cultural reasons.

Data collection used both online and hard copy versions of the questionnaire. The online version, using Google Forms platform, was used to enhance convenience notably for mobile teams, while the hard copy version was used to mitigate potential internet connectivity issues.

### 2.6 Ethical Considerations

The study adhered with the ethical guidelines outlined in the Declaration of Helsinki. Participants were approached respectfully, with particular attention paid to cultural sensitivities, especially when recruiting female participants from households. Verbal informed consent was duly obtained before inclusion. Ethical approvals were obtained from the Institutional Review Board of Warwick University, UK, and the University of Constantine, Algeria. Specific authorizations were provided by the competent authorities of Boussemghoun.

### 2.7 Data Management and Analysis

All data was carefully transcribed from the hard copies to Google Forms. The final database was downloaded as an Excel sheet, coded and transferred to Jamovi, Version 2.3 for Windows (The jamovi project, 2022; https://www.jamovi.org) for data analysis.

Descriptive statistics were conducted to calculate mean and standard deviation (SD), or median and centiles (P75), for numerical variables as applicable, and frequencies (percentages) for categorical variables.

Experience and HCU scales were analyzed for internal consistency using Cronbach's alpha. Overall scores for each scale were calculated by summing the scores of their respective items. Spearman's correlation was used to analyze the correlation between HCU and Experience scores.

The level of HCU was categorized into ‘low’ and ‘high’ using the median HCU score as the cutoff point. The Mann‐Whitney U test was used to compare health satisfaction scores across HCU levels. Other factors associated with HCU were analyzed using chi square test.

Independent factors of high HCU were analyzed in two multivariate logistic regression models (Model 1 and Model 2), using the Backward Elimination method (based on Wald statistics). The models included all variables that showed significance in the bivariate analyses. Model 2 differs from Model 1 in that it used dummy variables for the following categories: age ≥ 60; widowed status; housewife or retired; household income > 100 K DZD; residency ownership. All statistical tests were 2‐sided, and a *p*‐value < 0.05 was considered statistically significant.

## 3 Results

### 3.1 Participants Flow and Missing Data Management

We received 412 participations, of which 264 (64.1%) were collected via Google Forms and the 148 (35.9%) via hard copies. Of the total, 400 were eligible, while the remaining 12 were not residents of Boussemghoun. Data missing was minimal and concerned only two variables: household income (5 observations) and residency mode (1 observation). The multiple imputation method, utilizing a single iteration, was employed to estimate the missing values.

### 3.2 Participants' Socioeconomic and Health‐Related Data

The sociodemographic and health‐related data of the 400 eligible participants are presented in Tables 1 and 2, respectively. Most remarkably, male ratio was high (2.48) and age revealed decreasing proportions from the youngest to oldest age categories. Majority of participants had very low (53.0%) or low (33.0%) household income. Regarding health‐related data, 67.0% reported having *El‐Chifa* insurance and 15.3% reported another health insurance. Chronic diseases were prevalent (39.7), with hypertension (15.8%) and diabetes (8.8%) being the most frequent. Notably, a high (36.5%) percentage of the participants declared having traveled for healthcare during the past year. Three‐quarters (73.3%) of respondents reported optimal satisfaction with their overall health status, defined as a score of 8 or higher out of 10.

**Table 1:** Sociodemographic determinants of healthcare utilization (*N* = 400).

Parameter	Level	Total, *n* (%)	Level of HCU		*p*‐value
			Low	High	
Sex	Male	285 (71.2)	235 (82.5)	50 (17.5)	
	Female	115 (28.8)	61 (53.0)	54 (47.0)	< 0.001
Age (years)	18–29	98 (24.5)	88 (89.8)	10 (10.2)	
	30–39	86 (21.5)	65 (75.6)	21 (24.4)	
	40–49	76 (19.0)	61 (80.3)	15 (19.7)	
	50–59	55 (13.8)	40 (72.7)	15 (27.3)	
	60 or older	85 (21.3)	42 (49.4)	43 (50.6)	< 0.001
Marital status	Single	152 (38.0)	130 985.5)	22 (14.5)	
	Married	218 (54.5)	153 (70.2)	65 (29.8)	
	Divorced	9 (2.3)	8 (88.9)	1 (11.1)	
	Widowed	21 (5.3)	5 (23.8)	16 (76.2)	< 0.001
No. Children	None	175 (43.7)	148 (84.6)	27 (15.4)	
	1–3	120 (30.0)	90 (75.0)	30 (25.0)	
	4+	105 (26.3)	58 (55.2)	47 (44.8)	< 0.001
Educational level	Illiterate	71 (17.8)	42 (59.2)	29 (40.8)	
	Primary	55 (13.8)	29 (52.7)	26 (47.3)	
	Middle school	88 (22.0)	67 (76.1)	21 (23.9)	
	Secondary	106 (26.5)	89 (84.0)	17 (16.0)	
	University+	80 (20.0)	69 (86.3)	11 (13.8)	< 0.001
Job status	Unemployed	70 (17.5)	60 (85.7)	10 (14.3)	
	Housewife	64 (16.0)	25 (39.1)	39 (60.9)	
	Active	223 (55.8)	186 (83.4)	37 (16.6)	
	Retired	43 (10.8)	25 (58.1)	18 (41.9)	< 0.001
Household income (DZD)†	Very low (≤ 30 K)	212 (53.0)	161 (75.9)	51 (24.1)	
	Low (30–60 K)	132 (33.0)	93 (70.5)	39 (29.5)	
	Average (60–100 K)	40 (10.0)	35 (87.5)	5 (12.5)	
	High (> 100 K)	16 (4.0)	7 (43.8)	9 (56.3)	0.005
Residency mode†	Ownership	185 (46.3)	120 (64.9)	65 (35.1)	
	Family house	188 (47.0)	155 (82.4)	33 (17.6)	
	Rental	22 (5.5)	17 (77.3)	5 (22.7)	
	Other	5 (1.3)	4 (80.0)	1 (20.0)	0.002

^†^ Multiple imputation method was used to manage missing data for household income (5 observations) and residency mode (1 observation).

DZD: Algerian Dinar (1 DZD = US$ 0.0074).

All statistical tests are two‐sided.

**Table 2:** Health‐related determinants of healthcare utilization (*N* = 400).

Parameter	Level	Total *n* (%)	Level of HCU		*p*‐value
			Low *n* (%)	High *n* (%)	
*El‐Chifa* insurance Card holder	No	128 (32.0)	112 (87.5)	16 (12.5)	
	Yes	268 (67.0)	180 (67.2)	88 (32.8)	
	Do not know	4 (1.0)	4 (100.0)	0 (0.0)	< 0.001
Other health insurance	No	339 (84.8)	251 (74.0)	88 (26.0)	
	Yes, private	47 (11.8)	35 (72.3)	13 (27.7)	
	Yes, other	14 (3.5)	11 (78.6)	3 (21.4)	0.896
Smoking status	Nonsmoker	258 (64.5)	181 (70.2)	77 (29.8)	
	Ex‐smoker	49 (12.3)	38 (77.6)	11 (22.4)	
	Current smoker	93 (23.3)	77 (82.6)	16 (17.2)	0.049
No. chronic diseases†	None	241 (60.3)	207 (85.9)	34 (14.1)	
	1	103 (25.8)	66 (64.1)	37 (35.9)	
	2+	56 (14.0)	23 (41.1)	33 (58.9)	< 0.001
Number of chronic medications	None	301 (75.3)	252 (83.7)	49 (16.3)	
	1	37 (9.3)	21 (56.8)	16 (43.2)	
	2+	62 (15.6)	23 (37.1)	39 (62.9)	< 0.001
COVID‐19 during past year	No	337 (84.3)	249 (73.9)	88 (26.1)	
	Yes	35 (8.8)	23 (65.7)	12 (34.3)	
	Not sure	28 (7.0)	24 (85.7)	4 (14.3)	0.179
Surgery during past year	No	368 (92.0)	282 (76.6)	89 (23.4)	
	Yes	32 (8.0)	14 (43.8)	18 (56.3)	< 0.001
Other serious health issue during past year	No	369 (92.3)	281 (76.2)	88 (23.8)	
	Yes	31 (7.8)	15 (48.4)	16 (51.6)	0.001
Hospitalization during past year	No	370 (92.5)	283 (76.5)	87 (23.5)	
	Yes	30 (7.5)	13 (43.3)	17 (56.7)	< 0.001
Traveled for healthcare during the past year	No	254 (63.5)	217 (85.4)	37 (14.6)	
	Yes	146 (36.5)	79 (54.1)	67 (45.9)	< 0.001
Perceived overall health status	Suboptimal (< 8/10)	107 (26.8)	64 (59.8)	43 (40.2)	
	Optimal (≥ 8/10)	293 (73.3)	232 (79.2)	61 (20.8)	< 0.001

HCU: Healthcare utilization level, with low and high levels defined as HCU score < 9 and ≥ 9 respectively, and the cutoff value corresponds to the median value of the score variable.

^†^ The most frequent chronic diseases include hypertension (15.8%), diabetes (8.8%), respiratory diseases (8.3%), dyslipidemia (6.3%), thyroid dysfunction (4.8%), and rheumatic diseases (3.8%).

All statistical tests are two‐sided.

### 3.3 Experiences With Local Healthcare Services

Experiences with healthcare services showed positive feedback on most items, such as ease of accessibility (69.3%) and availability (63.3%) of local healthcare services, reliability of transportation to healthcare facilities (75.3%), and ease of contact with vital health services (76.8%). The alignment of services with personal choices (61.5%), educational or cultural backgrounds (71.3%), and respect of confidentiality (73.0%) also received positive feedback. However, only 18.8% agreed that their area contains all needed healthcare services, 57.6% found waiting times reasonable and comfortable, and less than half (48.3%) found the financial costs reasonable.

Reliability analysis of the Experience scale showed a Cronbach's alpha of 0.82 (10 items); and the mean overall experience score was calculated at 36.31 out of 50 (SD = 8.48). Results for this section are not presented in tables.

### 3.4 Spatial Disparities

The majority of participants (86.5%) reported a travel time of less than 15 min to the nearest care center, with 86.8% living within 5 kilometers of these facilities. Nonetheless, 7.5% indicated significant geographical barriers and 8.3% reported significant infrastructural challenges in accessing these facilities (Results not tabulated).

### 3.5 Levels of Healthcare Utilization

**Figure 1:**
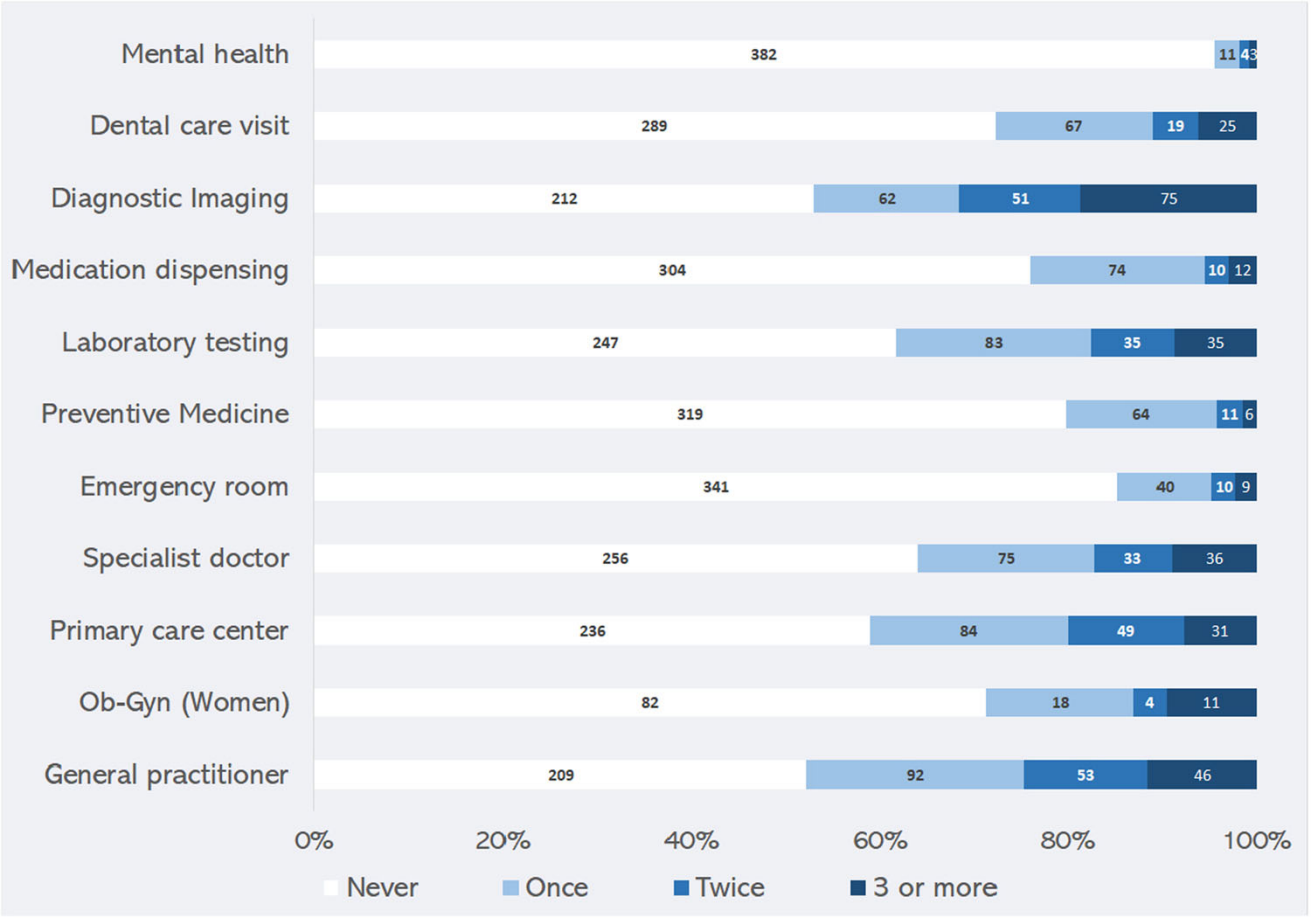
Levels of healthcare utilization of 11 basic care services during the past 12 months, among adult residents of Boussemghoun, Al Bayedh Province, Algeria, 2023.

The levels of utilization of the 11 care services are depicted in Figure 1. We observed a predominant trend of underutilization across all services, particularly in mental health, preventive medicine, and dental care. Similarly, ob‐gyn services were utilized by only 29% of women. Furthermore, 52.3% of the participants never visited a general practitioner during the past year. Cronbach's alpha of HCU scale was 0.74 (10 items: ob‐gyn was combined with specialist doctor) indicating reliability of the scale. The median (P75) HCU score was 4 (9) out of 33. Thus, high HCU was defined as HCU score ≥ 9.

### 3.6 Socioeconomic and Health‐Related Factors of HCU

Significant (*p* < 0.05) disparities in HCU were observed across all studied socioeconomic factors (Table 1). Additionally, participants who had *El‐Chifa* card, those with chronic diseases, and those taking chronic medications were more likely to have high HCU compared to their counterparts, and the differences were statistically significant. High HCU was also more common in individuals who reported surgery, other serious health issues, or hospitalization in the past year, compared to their respective counterparts. Additionally, participants who traveled for healthcare or perceived their health as suboptimal also showed higher HCU compared to their counterparts respectively. Other significant factors are detailed in Table 2.

### 3.7 Effect of Experiences and Spatial Disparities

No correlation was observed between HCU and Experience scores (Spearman's rho = −0.03, *p* = 0.576). Longer travel times (> 30 min) to local facilities were associated with a greater probability of high HCU (44.8% *vs.* 24.5%, *p* = 0.016) compared to shorter travel times, respectively. However, travel distances, geographical barriers, and infrastructural showed no significance (Results not tabulated).

### 3.8 Independent Factors of High HCU

The multivariate Model 1 identified 7 independent factors, explaining 28.5% of the variance in HCU. These factors included being female (OR = 2.62, *p* = 0.003), widowed status (OR = 4.65, *p* = 0.026), high household income (OR = 3.64, *p* = 0.048), taking 2 or more chronic medications (OR = 3.61, *p* = 0.001), experiencing other serious health issues in the past year (OR = 3.19, *p* = 0.013), travel to other regions for healthcare (OR = 1.87, *p* = 0.041), and long travel times to local facilities (over 30 min) (OR = 3.84, *p* = 0.004). Besides, housewife or retired status (OR = 2.22, *p* = 0.019) was significant in Model 2. Detailed results are presented in Table 3.

**Table 3:** Independent factors associated with high healthcare utilization.

Predictor	Level	OR	95% CI		*p*‐value
**Model 1**					
Sex	Female	2.62	1.40	4.90	0.003
Marital status	Single	Ref	—	—	0.042
	Married	1.59	0.82	3.08	0.173
	Divorced	0.20	0.02	2.33	0.199
	Widowed	4.65	1.20	18.00	0.026
Household income (DZD)	≤ 30 K	Ref	—	—	0.051
	30–60 K	1.19	0.66	2.16	0.565
	60–100 K	0.37	0.11	1.17	0.091
	> 100 K	3.64	1.01	13.08	0.048
*El‐Chifa* Card holder†	Yes	1.93	0.96	3.90	0.065
Number of chronic medications	None	Ref	—	—	0.002
	1	1.90	0.78	4.64	0.159
	2+	3.61	1.74	7.52	0.001
Other serious health issue†	Yes	3.19	1.28	7.91	0.013
Traveled for healthcare†	Yes	1.87	1.03	3.41	0.041
Long travel time (> 30 min)†	Yes	3.84	1.52	9.71	0.004
**Model 2**					
Sex	Female	1.90	1.00	3.61	0.049
Widowed‡	Yes	2.76	0.84	9.04	0.093
Housewife or retired‡	Yes	2.22	1.14	4.32	0.019
High‐income household‡	Yes	4.27	1.20	15.13	0.025
El‐Chifa card holder	Yes	1.96	0.99	3.86	0.052
Number of chronic medications	None	Ref	—	—	0.020
	1	1.75	0.73	4.21	0.213
	2+	2.92	1.37	6.27	0.006
Other serious health issue†	Yes	2.95	1.19	7.28	0.019
Traveled for healthcare†	Yes	1.79	0.98	3.27	0.057
Long travel time (> 30 min)†	Yes	4.06	1.62	10.22	0.003

Both models used multivariate logistic regression – dependent variable: high healthcare utilization; variable entry method: Backward Elimination (Wald). Model 1 yielded 11 steps while Model 2 yielded 10 steps. Model 1 and Model 2 explained 28.5% and 25.2% of the dependent variable's variance (Cox & Snell R Square = 0.285 and 0.252) respectively.

Variables excluded from Model 1 equation: age group (excluded at Step 11), number of children, educational level (excluded at Step 8), job status, residency ownership, number of chronic diseases (excluded at Step 4), surgery during past year (excluded at Step 5), hospitalization during past year, and health satisfaction level (excluded at Step 6).

OR: Odds Ratio; 95%CI: 95% confidence interval; Ref: Reference category for OR calculation.

^†^ OR calculated relative to the ‘no/none’ category.

^‡^ dummy variable was used.

## 4 Discussion

### 4.1 Summary of Findings

This study assessed healthcare access in a remote Algerian village, by quantifying HCU and analyzing interindividual disparities based on socioeconomic and health factors, spatial disparities and experience with healthcare services. Findings showed an overall low HCU across all care services, contrasting with high potential needs. High HCU was associated to various sociodemographic and health‐related factors, notably higher income which was significant in both multivariate models. Experience with local healthcare services showed a critical unmet need for a comprehensive care, associated with frequent traveling for healthcare and significant healthcare expenditures among the residents. While spatial disparities were minimal in the village, travel time was a significant predictor of high HCU.

### 4.2 Inconsistency of HCU Levels With Health Needs

Over a 12‐month period, none of the 11 healthcare services achieved 50% of utilization rate. In line with our findings, several studies showed low HCU levels in rural populations compared to urban ones, reporting concerns of physical accessibility, financial resources, and travel distance, besides inadequate care‐seeking behavior [15, 16, 17].

Nonetheless, the relatively low HCU observed contrasts with the high healthcare needs suggested by the prevalent self‐reported morbidity. We observed a significant burden of chronic diseases affecting approximately 40% of the participants, in addition to a high incidence of acute conditions in the past year, adding to the potential health needs. The inconsistency between HCU and health needs is further demonstrated by the absence, in the multivariate analysis, of an independent association of high HCU with chronic diseases or health satisfaction level. This suggests inequitable utilization of healthcare services based on needs.

According to the Andersen health behavior model, equity in health is achieved when HCU is strongly predicted by the needs. This widely accepted model categorizes HCU determinants into “predisposing factors” (such as sociodemographic factors), “enabling factors” (such as financial resources and physical accessibility), and “need factors” such as perceived health status and chronic diseases [13, 18]. An interesting study using this model in an agricultural province of China showed that having a chronic disease independently increased physician visit and hospitalization by 5.9‐ and 4.0‐fold, respectively. On the other hand, the impacts of predisposing and enabling factors were inconsistent and less significant [19]. In contrast, our study showed that HCU was not associated with chronic diseases in the multivariate model, while taking 2 or more medications and experiencing a serious acute condition were significant. This suggests that critical aspects of chronic disease management, such as regular doctor visits and comprehensive care, may be underutilized or inaccessible. It also reflects adverse health‐seeking behavior, relying mainly on acute care and showing poor adherence to continuity of care.

Beyond access and needs, effective HCU depends on several patient‐related factors, such as the ability to perceive these needs and to seek care [20]. These 2 dimensions are influenced by various factors including health literacy, awareness, perceptions and beliefs, and the use of informal care practices such as traditional remedies [20, 21]. Such dimensions were not explored in the present study, which could have provided valuable insights into addressing ineffective HCU.

Another remarkable finding is the low level of utilization of gynecology services, which achieved a rate of 29.7%. Consulting a women's health specialist can be motivated by various non‐illness reasons, such as family planning, mammogram screenings, prenatal care, etc. However, the utilization of such care services may be impeded by limited health autonomy for women, a prevalent issue in several developing countries, influenced by various socioeconomic and cultural factors [22].

### 4.3 Need for Comprehensive Services

This study emphasized the interconnectedness of the lack of comprehensive care, interregional healthcare mobility, and burden of healthcare costs as a triad that perpetuates inequality in health access. This is demonstrated by the high percentage of participants who traveled for healthcare during the past year, along with the lowest agreement to the Experience item addressing the availability of all needed healthcare services locally. Additional analysis showed that these two dimensions were significantly associated (Mann–Whitney *U* test, *p* < 0.05). Moreover, experiences showed the financial burden as the second major concern among the participants.

The absence of comprehensive care services and trained or specialized healthcare providers is a prevalent issue in rural areas [8]. In such circumstances, traveling to other regions – in search of better care – is associated with substantial costs, ineffective care utilization and poorer health outcomes [4, 5]. In progressive diseases, reliance on distant care facilities exposes to the risk of delayed diagnosis, reduced adherence to care, and increased mortality [6, 7]. Additionally, the necessity to travel for better care further widens the inequality gap based on financial resources, as supported by findings of the present study.

To tackle this issue, it is imperative to strategically enhance access to comprehensive care by prioritizing specialties and services that align closely with the specific health needs and prevalent morbidities among village residents. A data‐driven approach is necessary to assess these needs, incorporating an understanding of the factors driving residents to travel for healthcare. Such an approach ensures that resource allocation is need‐based and mitigates unnecessary travel and financial burdens currently faced by residents. This solution requires efficient strategies to recruit and retain competent healthcare professionals. Strategies to encourage rural practice include rural training opportunities and financial or other incentives [23, 24]. Antecedents of practice in rural areas were demonstrated to enhance professional's sensitivity to community needs [25]. Another cost‐effective and sustainable solution is telehealth, which has proven effective in improving care quality in rural settings and reducing travel and healthcare costs, besides other applications such as self‐management and professionals' training [26, 27].

### 4.4 Economic Status as a Major Enabling Factor

We found high income as one of the major determinants of high HCU, showing significance in both multivariate models. This, combined with the absence of significance for chronic diseases and health satisfaction, denotes the disadvantage of low‐income families in reaching adequate care for comparable needs. Socioeconomic deprivation is recognized compound inadequate health access in remote areas [28]. This is consistent with the Anderson health behavior model, showing income as an enabling factor for HCU [18]. Furthermore, most of the participants belonged to low or very low economic classes, emphasizing the significance of this issue in the village.

### 4.5 Effect of Other Socioeconomic Factors

While females were underrepresented, their HCU was significantly higher than that of males. Gender disparities in healthcare uptake are commonly reported, notably in rural areas. However, the extent of these disparities varies across regions and type of healthcare services, with various determinants influencing the direction of these gender differences [29, 30].

Unexpectedly, less educated participants showed higher HCU, though this was only significant in unadjusted analysis. This suggests that educational level did not act as an enabler for HCU as expected in the Andersen model [19]. The relationship between education and HCU is multifaceted. While higher education is associated with better health literacy and more effective HCU, lower education exposes to greater morbidity and lesser use of preventive care, resulting in greater reliance on curative care [31]. Understanding this dynamic is crucial for public health strategies, highlighting the need for tailored health education and outreach programs to enable effective HCU.

### 4.6 Effect of Healthcare Insurance

Health insurance coverage is a significant enabler of equitable health access, as it constitutes a financial protection for deprived individuals. It enhances healthcare services uptake and is associated with improved health outcomes and wellbeing and reduced out‐of‐pocket expenditures [32].

The Algerian public health insurance system is facilitated through *El‐Chifa* (“The Healing”, in Arabic) social security card, which allows 100% payment for prescriptions related to chronic diseases and a rationalized, third‐party payment for all other medical prescriptions [33]. As of 2022, *El‐Chifa* covered 31 million citizens [34], representing 69% of the total Algerian population and consistent with the 67% observed in the present study. Lately, government partnership with specialized private clinics extended to cover cardiovascular diseases, hemodialysis, and gynecology‐obstetrics care [9, 34].

However, despite insurance coverage, the lack of comprehensive care delivery can lead to persistent inequalities in accessing certain healthcare services, disadvantaging low‐income individuals [35]. This is consistent with our findings showing that having *El‐Chifa* card did not independently predict high HCU, after adjusting for income and other parameters. This further underscores the need for comprehensive local care to enhance equitable access.

### 4.7 Spatial Disparities

Spatial disparities were minimal, with no notable influence on HCU, except travel time to the nearest facility, which paradoxically correlated with higher HCU. In Boussemghoun, geographical barriers primarily affect nomadic tribes traveling across desert lands to reach the village center. This association may reflect greater health needs among nomadic populations due to delayed care‐seeking and poor adherence [36]. However, this study did not identify participants as nomads, limiting subgroup analysis.

A systematic review among African nomadic pastoralists showed HCU is shaped by geography, health literacy, culture, and sociopolitical factors [37]. Therefore, improving healthcare access among these populations involves tailored and culturally sensitive care delivery methods. These may include combining fixed and mobile services reinforced with strong awareness and educational actions [38]. It is important to note that the nomads of Boussemghoun tend to remain in the village peripheries. This semi‐nomadic lifestyle presents an opportunity for targeted, community‐informed, and sustainable healthcare interventions.

### 4.8 Implications for Action

The synthesis of these findings and their implications suggest a set of actions to enhance health access in Boussemghoun and comparable villages in Algeria.

- The key approach appears to be the implementation of comprehensive care delivery within the village, with a strategic plan to recruit and retain key health specialists [23, 24, 25].
- Training of local health professionals for the management of specific chronic diseases might also be a cost‐effective action [39].
- Both previous actions can be supported by telehealth technology, fostering virtual multi‐disciplinarily and referral system [26, 27].
- Patient‐targeted educational programs may also be effective in improving health literacy and care‐seeking behavior, thereby promoting preventive care, health empowerment and efficient HCU [40].
- The overall strategy should be evidence‐based, prioritizing urgent needs and tailoring actions to the sociodemographic and cultural specificities of the village, with plans for reassessment 3–5 years after implementation to evaluate long‐term effects on healthcare utilization and accessibility.
- Furthermore, replication of this study in other villages is recommended to assess similarities and contextual differences, allowing for the refinement of strategies based on comparative insights.

### 4.9 Strengths and Limitations

One of the strengths of this study is its sample size encompassing 8% of the total village population. Nevertheless, the sample representativeness might be affected by the convenience sampling method employed. This issue was partially addressed through the deployment of mobile data collection teams. Another limitation is the cross‐sectional design impeding the establishment of causal relationships between the different factors and HCU, along with introducing a recall bias due to the nature of the study questions. The self‐reported data may also impact the validity of the findings. Lastly, the generalization of these findings to other villages should consider the specific geographic obstacles and other characteristics that may variably influence access to health services.

## 5 Conclusion

Despite an apparent high need for healthcare in Boussemghoun, indicated by prevalent chronic and acute conditions, HCU among residents remains low. This is compounded by the critical lack of comprehensive care and the significant financial burden consequential to frequent interregional healthcare mobility. This exacerbates the inequalities in terms of access based on socioeconomic status and shapes an adverse care‐seeking behavior.

These findings underscore the urgency of enhancing comprehensive healthcare services within the village, focusing on specialties and services that align with the residents' specific health needs. This approach should include strategies for recruiting and retaining healthcare professionals, leveraging telehealth technologies, and implementing educational programs to promote health empowerment and efficient HCU. Additionally, considering the unique needs of nomadic tribes and incorporating their perspectives in healthcare planning is vital.

## Author Contributions

Conceptualization: Mohamed Amine Haireche, Md. Golam Kibria, Dr. Ali Davod Parsa, Dr. Russell Kabir. Data curation: Mohamed Amine Haireche. Formal analysis: Mohamed Amine Haireche. Investigation: Mohamed Amine Haireche. Methodology: Mohamed Amine Haireche, Dr. Ali Davod Parsa, Dr. Russell Kabir, Md. Golam Kibria. Supervision: Dr. Ali Davod Parsa, Dr. Russell Kabir. Visualization: Mohamed Amine Haireche. Writing – original draft: Mohamed Amine Haireche, Dr. Ali Davod Parsa, Dr. Russell Kabir, Md. Golam Kibria. Writing – review and editing: Mohamed Amine Haireche, Dr. Ali Davod Parsa, Dr. Russell Kabir, Md. Golam Kibria. All authors have read and approved the final version of the manuscript. All authors have read and approved the final.

## Conflicts of Interest

The authors declare no conflicts of interest. Specifically, no external funding sources or financial relationships were involved in the study design, data collection, analysis, interpretation, report writing, or the decision to submit the manuscript for publication.

## Transparency Statement

The lead author Md. Golam Kibria affirms that this manuscript is an honest, accurate, and transparent account of the study being reported; that no important aspects of the study have been omitted; and that any discrepancies from the study as planned (and, if relevant, registered) have been explained.

## Supporting information

Research Questionnaire.

## Data Availability

The data underlying this article will be shared on reasonable request to the author.
