# Supplementary material for: Assessing Healthcare Access in Rural Algeria: A Survey‐Based Cross‐Sectional Analysis of Healthcare Utilization Based on Socioeconomic and Health Factors, Experiences, and Spatial Disparities
Source: Health Sci Rep. 2025 Nov 23;8(11):e71565. doi: 10.1002/hsr2.71565 (PMC12641105; doi:10.1002/hsr2.71565)
Supplement: Supplementary file 1 — Research Questionnaire. [file HSR2-8-e71565-s001.pdf]

APPENDIX A – Research Questionnaire

Data Collector Code	C			SERIAL NUMBER			

Part A: Your Socioeconomic Data

A01	Sex	1○ Male 2○ Female
A02	Age	……years
A03	Marital Status	1○ Single 2○ Married 3○ Divorced 4○ Widowed
A04	Number of Children	……….. children
A05	Educational level	0. Illiterate 1 ○ Primary 2 ○ Middle School<br>3. Secondary 4 ○ University 5 ○ Postgraduate
A06	Professional status	0. Unemployed 1 ○ Housewife 2 ○ Employed<br>3. Self-employed 4 ○ Student 5 ○ Retired
A07	Spouse's professional status	0. Unemployed 1 ○ Housewife 2 ○ Employed<br>3. Self-employed 4 ○ Student 5 ○ Retired
A08	Household monthly income	1○ <30K DZD 3 ○ 30K – 60K DZD<br>2○ 60K - 100K DZD 4 ○ > 100K DZD
A09	Residency mode	1○ Ownership 2○ Rental 3○ With parents 4○ Other: ……

Part B: Your Health-related data

B01	Al Shifa Card holder	0○ No	1○ Yes	
B02	Other health Insurance	0○ None	1○ Yes, private	2○ Yes, other
B03	Smoking Status	1○ Nonsmoker	2○ Past smoker	3○ Current smoker
B04	Do you have any of these chronic diseases (Tick all that applies)	□ Thyroid disorders<br>□ Diabetes<br>□ High blood pressure	□ Respiratory diseases<br>□ Arthritis<br>□ High cholesterol / lipids	□ Mental illness<br>□ Cancer<br>□ Other
B05	No. chronic medications	1○ None	2○ One medication	3○ Two or more
B06	COVID-19 infection during the past 12 month	0○ No	1○ Yes	
B07	Surgical procedure during the past 12 months	0○ No	1○ Yes	
B08	Serious illness during the past 12 months	0○ No	1○ Yes	
B09	Hospitalization during the past 12 months			
B10	Other significant health issue during the past 12 months	0○ No	1○ Yes	
B11	Overall satisfaction from your current health status	………. / 10 (0=extremely unsatisfied, 10= extremely satisfied)		

Part C: Your Experience with Healthcare Services

Based on your personal experiences with healthcare services in your region, please indicate your level of agreement with each statement.
Please select: 1 = disagree; 2 = somewhat disagree; 3 = neutral; 4 = somewhat agree; 5 = agree.

C01	The healthcare services and resources I need are conveniently located within my region.	1□	2□	3□	4□	5□
C02	The healthcare services in my region are easy to reach.	1□	2□	3□	4□	5□
C03	The healthcare services are readily available when I need them.	1□	2□	3□	4□	5□
C04	Transportation to healthcare facilities is reliable and accessible.	1□	2□	3□	4□	5□
C05	The waiting times at healthcare services are reasonable and convenient.	1□	2□	3□	4□	5□
C06	The healthcare services respect my language and cultural background.	1□	2□	3□	4□	5□
C07	I can readily access contact details for crucial healthcare services like ambulances, my family doctor, and the hospital.	1□	2□	3□	4□	5□
C08	The healthcare services align with my personal needs and choices.	1□	2□	3□	4□	5□
C09	The financial costs of healthcare services are manageable and do not pose a significant burden on my or my family's budget.	1□	2□	3□	4□	5□
C10	My interactions with healthcare services have been positive, characterized by trust, respect, confidentiality, and cultural sensitivity.	1□	2□	3□	4□	5□

Part D: Spatial Disparities

Answer the following questions by choosing the option that is most appropriate to your specific case:

**D01**	How much time, in minutes, does it usually take you to travel from your home to the nearest primary care facility?	**<15 min**	**15-30 min**	**30-60 min**	**1-2 h**	**>2 h**
**D02**	What is the approximate distance, in kilometer, from your home to the nearest primary care facility?	**<5km**	**5-10 km**	**10-50 km**	**50-10 km**	**>100 km**
**D03**	To what extent do geographical barriers (e.g., mountains, rivers) obstruct your travel to the nearest primary care facility?	**1 = not at all**	**2 = slightly**	**3 = modera-tely**	**4= very much**	**5 = extre-mely**
**D04**	To what extent does the condition of transportation infrastructure (e.g., roads, bridges) negatively impact your access to the nearest primary care facility?	**1 = not at all**	**2 = slightly**	**3 = modera-tely**	**4= very much**	**5 = extre-mely**

Part E: Your Levels of Healthcare Utilization

Over the past 12 months, how many times have you experienced each of the following:

**E01**	General practitioner visit	**0.**□ Never	**1.**□ 1-2 times	**2.**□ 3 times or more
**E02**	Obstetrics / Gynecology*	**0.**□ Never	**1.**□ 1-2 times	**2.**□ 3 times or more
**E03**	Primary care center visit	**0.**□ Never	**1.**□ 1-2 times	**2.**□ 3 times or more
**E04**	Specialist doctor visit	**0.**□ Never	**1.**□ 1-2 times	**2.**□ 3 times or more
**E05**	Emergency room visit	**0.**□ Never	**1.**□ 1-2 times	**2.**□ 3 times or more
**E06**	Preventive screenings and vaccinations	**0.**□ Never	**1.**□ 1-2 times	**2.**□ 3 times or more
**E07**	Laboratory testing and bloodwork	**0.**□ Never	**1.**□ 1-2 times	**2.**□ 3 times or more
**E08**	Medication dispensing and prescription refills	**0.**□ Never	**1.**□ 1-2 times	**2.**□ 3 times or more
**E09**	Diagnostic Imaging (X-ray, CT scan, etc.)	**0.**□ Never	**1.**□ 1-2 times	**2.**□ 3 times or more
**E10**	Dental care visit	**0.**□ Never	**1.**□ 1-2 times	**2.**□ 3 times or more
**E11**	Mental health counseling	**0.**□ Never	**1.**□ 1-2 times	**2.**□ 3 times or more

*Female only.

Thanks for your participation

Note: The questionnaire was administered in the Arabic language, with respect of the cultural and linguistic specificities of the target population.
